# Current status and influencing factors of test anxiety of senior one students in Yanji, China: a cross-sectional study

**DOI:** 10.3389/fpsyg.2024.1414215

**Published:** 2024-07-23

**Authors:** Xin-Yang Xing, Gui-Meng Wang, Ying Li, Wen-Xuan Zhang, Xiang-Dan Shen

**Affiliations:** ^1^School of Nursing, Yanbian University, Yanji, Jilin, China; ^2^Department of Psychology, Yanbian Brain Hospital, Yanji, Jilin, China; ^3^School of Medicine, Anhui University of Science and Technology, Huainan, Anhui, China

**Keywords:** first year senior high school students, test anxiety, Yanji region, mental health, personality traits

## Abstract

**Objective:**

Examining the current situation of test anxiety among first year senior high school students in Yanji City and investigating the factors that contribute to exam anxiety.

**Methods:**

Using cluster sampling, a survey was conducted on 1,550 first-year high school students from three high schools in Yanji City in April–May 2023. The survey utilized general information questionnaires, the Minnesota Multiphasic Personality Inventory (MMPI), and the Self-Rating Anxiety Scale (SAS). Logistic regression analysis was used to determine the influencing factors of test anxiety.

**Results:**

A total of 1,550 first-year high school students were included in the analysis, with a test anxiety occurrence rate of 79.8%. Test anxiety exhibited statistical differences among different genders, ethnicities, family economic levels, frequency of communication with parents, and relationships with parents (with results of 53.44, 10.42, 17.31, 20.42, 31.95, all *p* < 0.05). Scores of hypochondriasis (Hs), depression (D), psychasthenia (Pt), paranoia (Pa), psychopathic deviate (Pd), schizophrenia (Sc), and hypomania (Ma) in the 10 clinical personality scales were significantly positively correlated. Logistic regression analysis revealed that gender, ethnicity, frequency of communication with parents, and scores of hypochondriasis (Hs), depression (D), psychasthenia (Pt), paranoia (Pa), and hypomania (Ma) in the 10 clinical personality scales were the main influencing factors for test anxiety in first-year high school students (all *p* < 0.05).

**Conclusion:**

The test anxiety level of high school students in Yanji City is relatively high, with variations in test anxiety levels among students of different genders, ethnicities, parental communication styles, and deviant personality traits. It is recommended that schools and teachers should give more consideration to test anxiety among high school students, particularly targeting those with potential risk factors. Parents should also be more attentive to their children’s development and advancement, and improve their family education principles.

## Introduction

With the development of society, people are encountering increasingly frequent and diverse pressures and anxieties in their lives. Stress and anxiety are non-specific reactions that can occur in the human body under any conditions (Onwuegbuzie, 2004). They are psychological conflicts that individuals experience in the interaction between themselves and their environment, such as reactions to illness and daily life troubles. They are also often expressed as negative factors when individuals cannot adapt to the requirements of the environment. Individuals will experience stress and anxiety when their requirements are frustrated by the environment and they are unable to respond appropriately (Onwuegbuzie, 2004; Ren, 2024).

Test anxiety is a common phenomenon that affects students of all ages and educational levels, with a prevalence rate between 41 and 55% (Krankenkasse, 2015; Tsegay et al., 2019; Vicent et al., 2023; Broks et al., 2024). Although test anxiety can be diagnosed as specific phobias or social anxiety disorder in the International Classification of Diseases (ICD-10) (Ortenburger, 2013), it does not have its own diagnostic category, resulting in difficulties regarding the reliability and comparability of scientific results. In general, test anxiety is a specific psychological response that occurs under the stimulating situation of exams, influenced by individual cognition, personality, and evaluation. It is typically characterized by worry and manifested through varying degrees of emotional reactions (World Health Organization, 2004; Zheng et al., 2022). Appropriate anxiety can stimulate learning motivation, while excessive test anxiety not only leads to lower grades (Huang and Zhou, 2019; Jiang et al., 2022) but also threatens mental and physical health (Bashir et al., 2019; Putwain et al., 2021; Mu et al., 2022) and can even develop into anxiety disorders. Globally, there have been numerous studies on the prevalence of test anxiety among adolescents, with rates varying by country. In India, it is estimated that two-thirds of students suffer from moderate to high levels of test anxiety (Lohiya et al., 2021), with around 8% (Ann Mary et al., 2014) to 18% (Koelen et al., 2024) having severe test anxiety disorders. In England, research shows that 16.4% of secondary school students report severe test anxiety (Putwain and Daly, 2014). However, studies from China indicate that the prevalence of severe test anxiety ranges from 30 to 46.7% (Shen et al., 2018; Li et al., 2024). Therefore, test anxiety is a common public health issue, with its prevalence generally believed to be on the rise (Chen et al., 2023).

In the context of the examination-oriented education system in China, coping with various exams has become a daily routine for Chinese students in their learning and life. Particularly for high school students, preparing for the upcoming college entrance examination (gaokao), which is considered the most pivotal event in their lives and may, to some extent, determine their future, can result in varying degrees of exam anxiety (DordiNejad et al., 2011). Recent studies have revealed that the average occurrence rate of exam anxiety among Chinese high school students is 37% (Wan et al., 2024). For freshmen in high school, the first year is a period of adjustment and preparation, during which they not only encounter heavy academic tasks but also need to adapt to new interpersonal relationships and environments. This adjustment period can easily lead to adaptation disorders, showing significant differences compared to students in the second and third years (Xu et al., 2022). Psychological adaptation and changes during the freshman year are crucial for the entire high school experience. Therefore, investigating the influencing factors and mechanisms of exam anxiety among high school freshmen is of great significance in addressing their exam anxiety issues and promoting their healthy development.

The generation of exam anxiety varies among individuals. Existing research shows that factors influencing exam anxiety in high school students include both external and internal factors (Wuthrich et al., 2020). The exam anxiety of first-year high school students is not only related to external factors (such as gender, curriculum reform, province, etc.) but also influenced by internal factors (personality, self-efficacy, personality traits, emotional regulation, etc.) and family factors (relationship with parents, family communication, etc.) (Sarason, 1984; Zeidner and Matthews, 2005; Putwain, 2008). In recent years, research on student groups has increasingly focused on the impact of internal and family factors on their exam anxiety (Liu et al., 2006). Students’ personality traits will affect their ability to adapt to the environment, indirectly influencing their level of exam anxiety (Li et al., 2009). Therefore, individual differences are the main reason for students to experience anxiety in the same exam situation. Existing research often analyzes and describes the high school stage as a unified whole and mostly uses convenience sampling methods, resulting in small sample sizes and biased research results. In addition, there are few reports on the impact of personality traits and family factors on exam anxiety in first-year high school students, confirming that these factors help reduce the occurrence of exam anxiety disorders. Last but not least, research on this topic in the Chinese context is very limited. China has a distinctive high school education system and is the largest developing country, with research results that can provide a reference for other countries and regions. Yanji City is located in the Yanbian Korean Autonomous Prefecture of Jilin Province, China (Wu, 2022). It is a border ethnic area and the capital city of the autonomous prefecture. The residents are Chinese Koreans, one of the cross-border ethnic groups in the northeastern border areas. Compared to other regions in China, the characteristics of Yanji City include lower levels of education and economy. After China’s reform and opening up, especially after the establishment of diplomatic relations between China and South Korea, Chinese Koreans have been moving to South Korea through forms such as visiting relatives, marriage migration, and labor export (Wang, 2019). This migration is not only large in scale and long in duration, but the number of people is increasing year by year. The remaining individuals are mostly teenagers and elderly people, leading to the disruption of high school students’ family structures and functions. This means that high school students in Yanji City may face more exam anxiety, personality traits, and family function issues. Therefore, this study takes all first-year high school students in Yanji City as the research subjects, aiming to investigate the current situation of their exam anxiety, analyze influencing factors, and provide a reference basis for helping first-year high school students smoothly adapt to the school and discover scientifically effective intervention measures.

## Methods

### Study design

A cross-sectional study was conducted and reported following the Strengthening the Reporting of Observational Studies (STROBE) statement (von Elm et al., 2007).

### Procedure and participants

From April to May 2023, a cluster sampling method was employed to select first-year high school students from three high schools in Yanji City (one Chinese Korean school and two Han Chinese schools) as the research subjects. The inclusion criteria were as follows: ① full-time first-year high school students; ② individuals without mental disorders, possessing normal hearing and vision, having no communication barriers; willing to participate in the study and sign an informed consent form; no severe cognitive impairments and capable of independently completing the tests. The exclusion criteria included: ① students on leave, suspended, dropped out, or unwilling to participate in the questionnaire survey; ② individuals who had taken part in similar studies in the past 3 months. The survey team comprised seven members, including three professionally qualified psychological assessors and four nursing master’s students. Prior to the survey, the investigators underwent one-on-one training, questionnaires were distributed by class, homeroom teachers assisted in assembling the subjects in classrooms, and the subjects were required to autonomously respond to the questionnaires and make independent judgments based on the actual situation. The data from the Minnesota Multiphasic Personality Inventory (MMPI) were gathered by three professionally qualified psychological assessors. A total of 1860 questionnaires (100%) were collected. After Following screening, MMPI three validity scales (F scale for faking bad, L scale for lying, Q scale for defensiveness) were first initially utilized quality control. Subsequently, invalid questionnaires were removed eliminated (comprising for faking bad, 63 for lying, and 4 for defensiveness), resulting in the inclusion of valid questionnaires (83.3%).

### Ethics statement

Ethical approval for this study was obtained from the ethics committee. The study was conducted in accordance with the Declaration of Helsinki. Informed consent was obtained from the school before collecting sample data. All students above 18 years old provided written informed consent. For students below 18 years old, informed consent was obtained from their legal guardians.

### Measurements

#### General information questionnaire

Researchers conduct studies by formulating their design through a comprehensive review of existing literature. This design is structured around the research objectives and content, encompassing two main components: general demographic characteristics and family status. General demographic characteristics consist of factors such as gender, age, ethnicity, living arrangements, and whether the individual is an only child. Family status, on the other hand, comprises elements like the economic status of the family, educational background of parents, frequency of communication with parents, and the quality of the relationship with parents.

#### Self-rating anxiety scale

In 1971, Zung (1971) developed a scale that was introduced to China by Duan and Sheng (2012). This scale can be used to assess the level of exam anxiety in students (Solomon and Rothblum, 1984). It consists of 20 items, divided into 4 levels of scoring based on the frequency of symptom occurrence, with 15 positively scored items and 5negatively scored items. The score is multiplied by 1.25 to obtain the standard score. According to the Chinese norm results (Zhang, 1998), the cutoff value for the SAS standard score is 50 points. A total score less than 50 indicates that the test subject may have anxiety issues, while a standard score < 50 is considered normal. Scores between 50 and 59 indicate mild anxiety, 60–69 indicate moderate anxiety, and ≥ 70 or higher severe anxiety. A higher score indicates a more severe level of anxiety.

#### Minnesota multiphasic personality inventory

In 1940, the MMPI (Minnesota Multiphasic Personality Inventory) was created by Hathaway and Mckinley (1940), professors at the University of Minnesota. Over time, the MMPI has undergone significant advancements and is now widely utilized as a psychological assessment tool across various disciplines, offering a comprehensive evaluation of subjects’ psychological traits (Song, 1989). This research incorporated three validity scales (Questioning-Q, Lying-L, Faking-F) and seven clinical scales (Psychopathic Deviate-Pd, Psychasthenia-Pt, Depression-D, Hysteria-Hs, Psychopathic Deviate-Pa, Schizophrenia-Sc, Hypomania-Ma), comprising a total of 168 items. As the participants in this study were minors, a simplified version of the MMPI was administered. It is important to note that direct comparisons cannot be made based on raw scores of each scale. According to the Chinese norm conversion standard, a T-score equal to or greater than 60 on each scale is considered abnormal, while a score of 70 or higher is deemed pathological. The reliability and validity of the Chinese version of the MMPI have been confirmed in previous studies (Song, 1985).

### Statistical method

Data were first checked manually for completeness, then coded and entered into Epi data version 3.1 and exported to IBM SPSS 26.0 software (IBM Corp., Armonk, NY, United States) version 21 for further analysis. The total scores and scores for each dimension of the SAS and MMPI tests were found to follow a normal distribution. Demographic information was presented as percentages, and the chi-square test was employed to compare exam anxiety levels among high school students with different characteristics. Spearman correlation and multiple-factor logistic regression were utilized to examine the relationship between personality traits and exam anxiety, as well as the factors influencing exam anxiety in students. Significance level is set at 0.05, where *p* < 0.05 indicates statistical significance. The variance inflation factor (VIF) was calculated to evaluate collinearity. Finally, the result was presented with texts and tables.

## Results

### Characteristics of the participants

This study included a total of 1,550 first year senior high school students, with 826 females (53.3%) and 724 males (46.7%), aged between 15 and 17 years old. See Table 1.

**Table 1:** Univariate analysis of test anxiety in the first year senior high school students.

Variables	Total *N* (%)	*χ^2^*	*p*
Gender			
Female	826 (53.3)	53.44	<0.001
Male	724 (46.7)		
Nation			
Chaoxian nationality	562 (36.3)	10.42	0.015
Han nationality	988 (63.7)		
Living situation			
Live with your parents	1,035 (66.8)	11.73	0.068
Live with the mother or the father	391 (25.2)		
Other	124 (8.0)		
Family economic level			
Upper	534 (34.5)	17.31	0.008
Medium	944 (60.9)		
Lower	72 (4.6)		
Only child			
Yes	922 (59.5)	2.52	0.471
No	628 (40.5)		
Education level of parents			
Junior high school and below	326 (21.0)	7.03	0.318
Senior high school	593 (38.3)		
Bachelor degree or above	631 (40.7)		
Frequency of communication with parents			
Always	866 (55.9)	20.42	0.015
Sometimes	538 (34.7)		
Rarely	134 (8.6)		
Never	12 (0.8)		
Relationship with parents			
Very good	843 (54.4)	31.95	<0.001
Better	537 (34.6)		
General	159 (10.3)		
Not good	7 (0.5)		
Very bad	4 (0.3)		

### Current situation of test anxiety level and personality traits

The study found that 79.8% of first year high school students exhibited symptoms of exam anxiety, with varying degrees of severity observed. Specifically, the prevalence rates of normal, mild, moderate, and severe anxiety were 20.1% (312 students), 49.4% (766 students), 28.7% (445 students), and 1.7% (27 students) respectively. Additionally, among the 1,128 high school students (72.8%) with scores higher than the Chinese norm on the 10 clinical scales of the MMPI, the distribution of characteristics is as follows: hypochondriasis (Hs) 514 students (33.2%), depression (D) 469 students (30.3%), psychasthenia (Pt) 279 students (18.0%), paranoia (Pa) 264 students (17.0%), psychopathic deviate (Pd) 253 students (16.3%), schizophrenia (Sc) 310 students (20.0%), and hypomania (Ma) 301 students (19.4%).

### Analysis of single factors contributing to test anxiety

There is no statistical difference in exam anxiety among high school freshmen based on different living situations, parents’ education levels, and being an only child. However, there are statistically significant differences in exam anxiety among high school freshmen based on gender (53.3% of females experience exam anxiety compared to 46.7% of males), ethnicity, family economic status, frequency of communication with parents, and the quality of relationships with parents (*p* < 0.05). See Table 1.

### The correlation between test anxiety and personality traits

The results of the analysis show a correlation between the personality traits of high school students and test anxiety. Specifically, the clinical scales Hs, D, Pt, Pd, Pa, Sc, and Ma in the MMPI are all significantly negatively correlated with test anxiety (*p* < 0.01). Please see Table 2 for more details.

**Table 2:** Association of factors with test anxiety.

Variables	Test anxiety	Hs	D	Pt	Pd	Pa	Sc	Ma
Test anxiety	1							
Hs	−0.301**	1						
D	−0.324**	0.416**	1					
Pt	−0.288**	0.380**	0.382**	1				
Pd	−0.240**	0.282**	0.268**	0.366**	1			
Pa	−0.270**	0.315**	0.281**	0.471**	0.436**	1		
Sc	−0.238**	0.319**	0.278**	0.458**	0.377**	0.486**	1	
Ma	−0.132**	0.084**	−0.004	0.122**	0.211**	0.211**	0.215**	1

^*^p < 0.05; ^**^p < 0.01.

### Multifactorial analysis of factors influencing exam anxiety

Using test anxiety as the dependent variable, a multiple logistic regression analysis was conducted with general demographic characteristics (gender, ethnicity, living situation, only child status, family economic status), family functioning (parents’ education level, frequency of communication with parents, relationship with parents), and personality traits (Hs, D, Pt, Pd, Pa, Sc, Ma scales) as independent variables. The entry and removal criteria were set at 0.05 and 0.10, respectively. The statistical results showed a good fit of the regression equation, with VIF values for both models being less than 10 and Tolerance values greater than 0.1, indicating no collinearity issues. The results indicated that gender, ethnicity, frequency of communication with parents, Hs, D, Pt, Pa, and Ma scales were the main influencing factors of test anxiety in high school students. Refer to Table 3 for detailed results.

**Table 3:** Logistic regression analyzes of test anxiety.

Variables	β	Waldχ^2^	*p*	OR(95%CI)
Gender				
Female	0.840	65.542	<0.001	2.32 (1.89 ~ 2.84)
Nation				
Chaoxian nationality	0.312	8.936	0.003	1.37 (1.11 ~ 1.68)
Family economic level				
Upper	0.065	0.067	0.796	1.07 (0.65 ~ 1.74)
Medium	0.231	0.918	0.338	1.26 (0.79 ~ 2.02)
Frequency of communication with parents				
Always	−1.295	4.261	0.039	0.27 (0.08 ~ 0.94)
Sometimes	−1.193	3.683	0.055	0.30 (0.09 ~ 1.03)
Rarely	−1.102	3.064	0.08	0.33 (0.10 ~ 1.14)
Relationship with parents				
Very good	1.506	2.053	0.152	4.51 (0.58 ~ 35.4)
Better	1.761	2.823	0.093	5.82 (0.75 ~ 45.33)
General	1.500	2.060	0.151	4.48 (0.58 ~ 34.78)
Not good	0.766	0.354	0.552	2.15 (0.17 ~ 26.82)
Hs				
Yes	0.588	22.786	<0.001	1.80 (1.42 ~ 2.29)
D				
Yes	0.802	39.651	<0.001	2.23 (1.74 ~ 2.86)
Pt				
Yes	0.646	15.338	<0.001	1.91 (1.38 ~ 2.64)
Pd				
Yes	0.291	3.139	0.076	1.34 (0.97 ~ 1.85)
Pa				
Yes	0.393	5.432	0.02	1.48 (1.07 ~ 2.06)
Sc				
Yes	0.290	3.501	0.061	1.34 (0.99 ~ 1.81)
Ma				
Yes	0.438	10.835	0.001	1.55 (1.19 ~ 2.01)

## Discussion

### It is quite common for first-year high school students to experience test anxiety

The investigation in this study reveals that the overall detection rate of test anxiety among first-year high school students in Yanji City is 79.8%, which is higher than the research findings of Ma (2010), Yu (2022), and Kim and Kim (2023) on Chinese Korean high school students. This disparity may be attributed to two main reasons. Firstly, the previous studies concentrated on high school students in general, while this study specifically focused on first-year students, resulting in variations in the prevalence of test anxiety. Secondly, the earlier studies only involved Chinese Korean high school students as participants, whereas this study’s first-year students encompass both Han and Korean ethnicities, with a ratio of approximately 2:1. The overall prevalence rate indicates that test anxiety among first-year students has become a prevalent issue. The analysis suggests that the students surveyed in this study have recently entered high school and are encountering changes in identity, psychology, learning approaches, and social circles (Wuthrich et al., 2021). Confronted with the rigorous academic demands of high school, students feel overwhelmed, anxious about the impact of exam failures on their academic performance, leading to poor mental well-being. Additionally, this may be closely related to China’s implementation of reforms in the examination and enrollment system (China State Council, 2014), with a stricter and more standardized selection of talents, emphasizing both academic performance and comprehensive student development. Students have just undergone the junior high school academic level test and are about to face the national unified college entrance examination, making test anxiety among first-year students more severe. This conclusion is consistent with Wang’s research findings (Wang, 2023).

The detection rates of mild, moderate, and severe test anxiety were 49.4, 28.7, and 1.7%, respectively. These rates were lower than the research findings of Tang (2023) but consistent with the study conducted by Lai et al. (2018). The proportion of moderate to severe anxiety is approximately one-third, indicating that high school students experience relatively severe test anxiety when dealing with the pressures of studying, exams, and further education. According to the stress theory model (Kalkhoven et al., 2020), when confronted with the stress of exams, high school students can either alter their perception of exams to better adapt to the external environment or suppress negative emotions such as anxiety, repression, and avoidance when feeling excessive pressure from exams. Research indicates that when stress is manageable, it can be beneficial for high school students academically (Xu et al., 2021), fostering a rational perspective on exam results, timely emotional regulation, and reduced anxiety levels. Therefore, schools can target high school students who struggle to express themselves and tend to suppress their emotions, encouraging them to broaden their perspectives on exam results to decrease the use of negative emotional regulation strategies like suppression and denial. This emphasizes the development of students’ healthy personalities and offers guidance on students’ mental health concerns. For students with moderate anxiety levels, further investigation into other physical discomforts can be conducted, and interventions can be determined based on additional clinical symptoms. Students with severe anxiety levels can receive active psychological guidance, including one-on-one counseling services. Tailoring the treatment of test anxiety based on its underlying causes and collaborating with parents and students can effectively address the test anxiety issues experienced by students. Furthermore, research has pointed out that poor mental states (Salehi et al., 2021), such as anxiety and depression, are important determining factors for the poor academic performance of children and adolescents. Exam anxiety is a significant modifiable factor affecting the health level of high school freshmen, which can impact students’ academic performance and mental health. Therefore, exam anxiety should not be seen as a normal behavior for high school freshmen. It is important to increase awareness of exam anxiety, strengthen guidance and management at the organizational level, and provide early psychological health education and counseling on exam anxiety during the high school entry stage to reduce exam anxiety levels.

### Current status of clinical typing of personality traits

The results of the MMPI clinical scale can be used to describe a person’s long-term stable personality traits, as well as to assess their current psychological state over a period of time, and the psychological changes under stress (Hsu et al., 2022). The top three personality trait clinical manifestations are somatization (Hs), depression (D), and dissociation from reality (Sc), which is similar to the results of Xu and Xu (2023). The reasons for the deviation in personality traits may be that students who exhibit somatization (Hs) and depression (D) are more prone to stress under academic pressure. They may have personality traits such as anxiety, depression, timidity, introversion, withdrawal, lack of confidence, relatively weaker responses to academic setbacks, increased sensitivity when facing difficulties, leading to frequent changes in physical discomfort symptoms, experiencing stronger anxiety emotions, and difficulty adapting to the surrounding environment. Students with dissociation from reality (Sc) personality traits in clinical manifestations are more likely to adopt negative coping strategies such as self-blame and fantasy when faced with stressful events. This significantly increases the stress, anxiety, and other negative emotions brought by the stressful events, thereby increasing the level of test anxiety. On the other hand, it may be due to the fact that first-year high school students are entering the high school stage, experiencing adaptive disorders, significant changes in psychology, learning methods, and social groups, leading to the emergence of clinical manifestations of personality traits such as somatization, depression, and dissociation from reality. Research has found that the developmental and stable nature of personality traits exhibit different manifestations at different stages of individual growth and are influenced by various external factors (Bleidorn et al., 2019). High school freshmen are in a critical period of adolescence, facing changes in identity, psychology, learning styles, and social groups, among other aspects. Their personality development is not yet mature, yet personality traits play a crucial role in their healthy growth. Therefore, it is recommended that schools and parents pay attention to the personality and psychological development of high school freshmen, focus on students who exhibit deviant personality traits during a period of time or under stress, and provide tailored treatment plans to help them develop towards a healthier direction physically and mentally. In addition to conventional prevention and treatment methods, schools and teachers need to help high school freshmen actively cope with stress, quickly adapt to high school life, establish positive ways to deal with new environments, provide emotional comfort, and offer social support.

### There is a correlation between test anxiety and personality traits in clinical manifestations

Although the results of MMPI assessments are not suitable for discussing psychiatric classifications, a wide range of clinical symptom assessments can provide references for future research. The results of this study indicate a negative correlation between personality traits clinical symptom scale and test anxiety in high school students. Deviant personality traits clinical symptoms such as hypochondriasis (Hs), depression (D), psychasthenia (Pt), paranoia (Pa), psychopathic deviate (Pd), schizophrenia (Sc), hypomania (Ma) are significantly positively correlated with test anxiety. Consistent with the results of Zhang and An (2016) and Pei et al. (2021), it indicates that deviant personality trait expressions can predict students’ levels of test anxiety. This may be because high school students with the above deviant personality traits clinical symptoms often exhibit abnormal emotional reactions and psychological symptoms, such as depression, delusions, distress, etc., leading to extreme and adverse emotional reactions. When facing adaptive difficulties such as heavy academic tasks, they are prone to test anxiety. Since personality trait expressions are closely related to the mental health of high school students, they may have a predictive effect on test anxiety (Cuijpers et al., 2021). It is suggested that schools and teachers should pay more attention to the types of personality traits clinical symptoms of high school students, conduct a general survey of personality traits upon enrollment, establish comprehensive information files for each student, focus on the clinical symptoms of personality traits of students, and pay special attention to students with abnormal personality trait expressions. On the other hand, high school freshmen have a relatively shallow understanding of mental health-related knowledge, lack a systematic grasp, and lack systematic learning of personality traits and mental health-related knowledge. This suggests that when schools organize and manage the development of mental health education courses, they need to focus on a combination of systematic and targeted approaches to promote high school freshmen’s cognitive recognition of personality traits.

### Pay special attention to high school freshmen who have potential risk factors and work on improving their family functioning

This study found that the frequency and deviation of parental communication in demographic data, as well as the personality traits of Hs, D, Pt, Pa, and Ma in clinical performance, are influencing factors for exam anxiety in high school students. The above research results can provide a basis for future prevention strategies and intervention measures for exam anxiety and personality traits in first-year high school students. The detection rate of exam anxiety in females is higher than in males, which is consistent with previous research results (Liu et al., 2021; Wang et al., 2021). The analysis suggests that the reasons may be due to the female body structure making them more susceptible to external influences, being more sensitive in emotional expression, having a lower threshold for emotional arousal, and a higher level of emotional arousal compared to males. Males have significantly stronger psychological resilience and emotional regulation abilities than females, and exhibit stronger defense mechanisms when acknowledging anxiety symptoms (Núñez-Peña et al., 2016). However, a study in the UK found that males have higher levels of exam anxiety than females, possibly due to cultural backgrounds and environmental differences between Eastern and Western cultures (Carey et al., 2017). In addition to the above factors, part of the reason may be attributed to the traditional Chinese belief of favoring males over females. With the reform of the new curriculum and the new college entrance examination in China, the distinction between arts and sciences is no longer made, and the high school stage emphasizes strong logic, higher requirements for spatial imagination and abstract thinking abilities, and a male advantage in mathematical and scientific subjects, making females more likely to experience anxiety from academic pressure. In terms of ethnic differences, Chinese Korean high school students are more prone to exam anxiety. The possible reasons for this could be influenced by traditional customs of the Chinese Korean ethnic group, where it is commonly believed that raising children is primarily the mother’s responsibility (accounting for 79.3%). In border areas of ethnic minority groups in China, the trend of Korean parents going out to work has led to changes in family structures, resulting in many students staying at home without parents being able to fulfill their responsibility of educating their children, leading to ineffective management of children and affecting their physical and mental development (He, 2010). In terms of the use of Chinese language ability, Korean students are constrained by factors such as the differences in language family between Korean and Chinese, as well as students’ cognitive abilities. There are still deficiencies in language knowledge and skills, as well as cognitive abilities (Rubin et al., 2018). These factors may lead to exam anxiety among Korean high school freshmen. Therefore, it is recommended that senior high schools in border areas focus on the characteristics of different groups when conducting mental health education for new high school freshmen, paying special attention to female and minority Korean students. Based on demographic characteristics and differences, targeted measures and plans should be developed to tailor exam anxiety relief and resolution programs.

Barnes and Olson categorized parent–child communication into open communication and problem communication (Lozano-Blasco et al., 2024). High school students’ exam anxiety is influenced by both problem communication and open communication in parent–child communication (Dai et al., 2018). The study’s findings indicate that the frequency of parental communication in family functioning affects high school students’ exam anxiety, aligning with Liu (2020) and Qian (2014) results. Some students experience reduced exam anxiety through frequent communication with their parents. The analysis suggests that certain students are willing and able to engage in clear conversations, discussions, and open emotional expressions with their parents. Open parent–child communication allows for comfortable information exchange and emotional expression, fostering shared perceptions and mutual understanding. The study emphasizes that the openness of parent–child communication should prevail over problems, thereby alleviating exam anxiety and psychological stress. However, increased communication frequency between some students and their parents can worsen exam anxiety. The analysis indicates that parents may adopt a problem communication approach, overly focusing on their children’s academic performance, impacting the students’ psychological well-being. Wu (2023) discovered that high school students, during interactions with their parents, tend to avoid discussing their inner feelings and thoughts or sharing much about themselves. When confronted with parental demands, high school students may exhibit annoyance and impatience. Moreover, inappropriate family environments and parenting styles are significant factors contributing to psychological issues in high school students (Dolz-Del-Castellar and Oliver, 2021; Xiu et al., 2022). Parents are advised to engage in positive emotional communication with their children based on their personality traits to reduce or prevent exam anxiety. They should proactively learn advanced family education concepts, establish a fair and democratic family environment, maintain appropriate communication frequency, provide children with sufficient independent space, consider their opinions in decision-making, comprehend their genuine needs, acknowledge their strengths and weaknesses, enhance family adaptability, build a relationship of mutual trust with their children, and encourage them to develop their own circle of friends. Children with poor emotional stability should be guided to acknowledge their emotions, analyze the reasons behind them, find solutions, collaborate with teachers to understand their school situation, and collectively create a healthy growth environment. Children with high neuroticism require special attention. In times of adversity, they need increased spiritual support, efforts should be made to stimulate their internal drive, unlock their inner potential, and nurture their drive for self-improvement.

### Limitations

However, this study also has some limitations. Firstly, as it is a cross-sectional study, it only captures the current period and reflects the exam anxiety and personality traits of high school students under stress. It cannot explore the impact of influencing factors on exam anxiety over time or establish causality. Secondly, although the MMPI is rigorously validated, its nature as a self-report test may introduce bias. The results of MMPI assessments are not suitable for discussing psychiatric classifications. Thirdly, due to limitations, only the MMPI was utilized for the initial screening of personality traits in clinical presentations. Finally, the study surveyed high school students from three senior high schools in Yanji City, which limits the regional representation in the sample. Therefore, it is recommended to conduct large-scale cohort studies targeting different regions and to combine MMPI assessments with other physiological indicators to accurately and comprehensively evaluate personality traits in clinical presentations. This approach will enable a more precise analysis of the relationship, influencing factors, and developmental patterns of exam anxiety and personality traits.

## Conclusion

In conclusion, the results of this study indicate that the level of exam anxiety among first-year high school students in Yanji City is relatively high. Factors influencing exam anxiety include gender, ethnicity, frequency of parental communication, and clinical manifestations of deviant personality traits. It is recommended that relevant education authorities should proactively address the mental health of first-year high school students and offer timely psychological interventions for students experiencing severe anxiety. Schools and teachers should increase their awareness of exam anxiety among first-year students, conduct regular follow-ups and evaluations for students with the mentioned characteristics to reduce their anxiety levels, and pay special attention to first-year students with potential risk factors. Strengthening family functioning, enhancing family education concepts, and promoting open communication between parents and children are necessary. Early prevention, detection, and diagnosis guidance for exam anxiety in first-year students should be reinforced to enhance their learning quality and support their healthy mental and physical development.

## Data availability statement

The raw data supporting the conclusions of this article will be made available by the authors, without undue reservation.

## Ethics statement

The studies involving humans were approved by Medical Ethics Review Committee of Yanbian University School of Medicine. The studies were conducted in accordance with the local legislation and institutional requirements. Written informed consent for participation in this study was provided by the participants’ legal guardians/next of kin.

## Author contributions

X-YX: Writing – original draft, Formal analysis, Software, Writing – review & editing, Investigation, Data curation. G-MW: Writing – original draft, Formal analysis, Software, Writing – review & editing, Investigation, Data curation. YL: Conceptualization, Methodology. W-XZ: Investigation, Data curation. X-DS: Supervision, Project administration, Validation.
